# Analysis of Combustion Behavior and Comparison of Kinetic Models of Oil Shale

**DOI:** 10.3390/molecules30081819

**Published:** 2025-04-18

**Authors:** Fajun Zhao, Hong Zhang, Yangming Liu, Lei Zhang, Sen Liu, Tianyu Wang, Meng Li

**Affiliations:** 1 Key Laboratory of Improving Oil and Gas Recovery, Northeast Petroleum University, Ministry of Education, Daqing 163318, China; zhanghonghong@petrochina.com.cn (H.Z.); r411971716@163.com (Y.L.); zl_alfred@126.com (L.Z.); lslddnb@163.com (S.L.); wtyabcc@163.com (T.W.); meng842019@163.com (M.L.); 2 State Key Laboratory of Continental Shale Oil, Daqing 163712, China; 3 Exploration and Development Research Institute of Daqing Oilfield Co., Ltd., Daqing 163712, China

**Keywords:** oil shale, combustion kinetics, thermogravimetric analysis (TGA), Ozawa–Flynn–Wall method, Kissinger method, multi-step reaction mechanism

## Abstract

This study examines the thermal characteristics and kinetics of oil shale combustion using thermogravimetric analysis (TGA) at various heating rates. The combustion process includes three stages: dehydration, main combustion (70–80% mass loss), and mineral decomposition. Kinetic analysis using model-free (Ozawa–Flynn–Wall, Kissinger) and model-based (multi-step reaction kinetics) methods revealed that the second-order reaction model (F2) had the highest accuracy. Oil shale combustion involves multi-step reactions, with activation energy and pre-exponential factors varying nonlinearly with conversion rates. Combining model-free and model-based methods provides insights for optimizing combustion processes and equipment design for the efficient utilization of unconventional energy resources.

## 1. Introduction

As a significant unconventional energy resource with abundant reserves and widespread distribution, oil shale has garnered considerable attention in recent years. It is an organic–inorganic composite sedimentary rock, primarily composed of inorganic minerals and organic matter that can be converted into oil and gas resources (i.e., “solid soluble organic matter”). Through processes such as pyrolysis, combustion, or gasification, oil shale can release usable thermal and chemical energy, offering a potential solution to replace traditional fossil fuels [1].

With the gradual depletion of conventional energy reserves, the development and utilization of unconventional energy resources have become critical areas of focus in the global energy sector. Against this backdrop, oil shale, with its resource abundance and potential, has emerged as one of the key priorities in unconventional energy development. However, the complex thermochemical behavior and multiphase reaction mechanisms involved in the combustion and pyrolysis of oil shale pose significant challenges to its efficient energy utilization [2]. Therefore, studying its combustion kinetics not only helps to elucidate key reaction mechanisms but also provides essential theoretical support for optimizing combustion processes and equipment design.

Kinetic studies are fundamental tools for understanding the combustion process of oil shale. However, the application of existing kinetic models in practice still faces numerous limitations. For example, the compositions of oil shale samples from different regions exhibit significant variability, which may lead to differences in combustion behavior and reaction characteristics, affecting the universality of the models. Additionally, variations in combustion conditions (such as oxygen concentration, particle size, and reaction temperature) pose further challenges to the applicability and accuracy of the models. More importantly, the parameter fitting process in kinetic models may be influenced by issues such as multiple solutions and experimental data deviations, which further restrict in-depth research and industrial application [3].

Current studies on the combustion characteristics of oil shale primarily rely on experiments using thermogravimetric analysis (TGA) and differential scanning calorimetry (DSC). Kok et al. used TGA-MS technology to study the pyrolysis and combustion characteristics of oil shale, revealing its multi-stage reaction process, including dehydration, organic matter pyrolysis, and mineral decomposition [4]. They also described in detail the behavior and gas product characteristics of each stage at different temperatures [4]. Liu et al. revealed the multi-stage characteristics of oil shale pyrolysis and combustion through thermogravimetric analysis Fourier transform infrared spectroscopy (TG-FTIR) technology and analyzed in detail the behavior and gas product characteristics of oil shale at various temperatures [5]. By analyzing the pyrolysis and combustion behavior under varying temperatures, heating rates, and atmospheric conditions, researchers have proposed various kinetic models, including single-step reaction models, multi-step reaction models, and models based on distributed activation energy theory [6,7,8,9]. These models describe the mass loss rate and reaction mechanisms of the oil shale combustion process from different perspectives, providing effective tools for analyzing and predicting experimental data.

However, these models still exhibit significant limitations in practical applications. Factors such as the diversity of sample compositions, the complexity of combustion conditions (e.g., variations in oxygen concentration and particle size), and issues like multiple solutions in parameter fitting all constrain the generality and depth of these models [10,11,12,13,14]. Li et al. emphasized the challenge of applying the kinetic model to the pyrolysis of oil shale and pointed out that the heterogeneous mineral composition and multi-stage reaction path made it complicated to select the appropriate kinetic framework [15]. Zhou et al., by combining model-free and model-based methods, demonstrated that the combustion dynamics of oil shale can be more comprehensively characterized, providing a scientific basis for combustion process optimization and equipment design [16]. Hua et al. used a distributed activation energy model (DAEM) combined with thermogravi-metric analysis (TGA) to study the pyrolysis kinetics of oil shale, revealing its multi-stage pyrolysis process, including volatile release, coke combustion, and mineral decomposition [17]. The kinetic parameters were calculated at different temperatures and heating rates, providing theoretical support for optimizing the pyrolysis process and equipment design [17]. Chen et al. used in situ Fourier transform infrared spectroscopy (FTIR) technology to study the evolution of hydrocarbon compounds during the pyrolysis process of oil shale, revealing their staged generation and transformation characteristics [18]. By monitoring the changes at different temperatures in real time, the evolution mechanism of hydrocarbon compounds was elucidated [18]. Chang et al. analyzed the influence of mineral matrix on the pyrolysis kinetics of oil shale through TGA, revealing the catalytic and inhibitory effects of mineral components during the pyrolysis process and their significant impact on pyrolysis kinetic parameters such as activation energy (Ea) and the pre-exponential factor (A), providing theoretical support for optimizing the pyrolysis process and improving energy utilization efficiency [19]. Therefore, it is imperative to develop accurate kinetic models capable of describing complex multi-step reactions to characterize the combustion process of oil shale comprehensively and provide a reliable theoretical foundation for its efficient utilization.

To address these challenges, this study systematically analyzes the kinetic characteristics of the oil shale combustion process by combining the advantages of model-free and model-based methods. The research not only reveals the key features of combustion mechanisms but also provides scientific support for the optimization of efficient combustion processes, holding significant scientific and engineering value.

## 2. Experimental Section

### 2.1. Materials and Sample Preparation

In this study, typical oil shale samples from the Daqing Oilfield were selected as the experimental objects. After crushing and sieving, particles with a size range of 0.25–0.5 mm were chosen for the experiments. The samples were dried at 105 °C for 24 h to remove moisture. The elemental composition and inorganic mineral content of the samples were determined using an elemental analyzer and X-ray diffraction (XRD), respectively. In addition, XRD and SEM analyses were conducted on the oil shale samples before and after combustion. In this study, the elemental analyzer used was produced by PerkinElmer (Waltham, MA, USA), with the PE 2400 Series II model; the SEM was manufactured by the FEI Company in the United States (Hillsboro, OR, USA), with the Quanta 450 FEG model; and the XRD was produced by Rigaku (Tokyo, Japan), with the Ultima IV model. The results of the elemental composition and inorganic mineral content of the samples are shown in Table 1.

### 2.2. Experimental Equipment and Methods

#### 2.2.1. Thermogravimetric Analysis (TGA)

The combustion characteristics of oil shale were measured using a thermogravimetric analyzer (TGA). The experiment was conducted on a NETZSCH TG209F3 thermogravimetric analyzer (NETZSCH, Selb, Germany), with the following experimental parameters: heating rates of 5, 10, 15, 20, and 25 °C/min; temperature range from room temperature to 1000 °C; reaction atmosphere of air (oxygen concentration 21%); and gas flow rate of 50 mL/min. For each experiment, a 5 mg sample was weighed and placed in a ceramic crucible, ensuring the experimental environment’s airtightness and stable gas flow. The TGA experiment recorded the relationship between sample mass and temperature, which was used to analyze the mass loss rate and reaction stages.

#### 2.2.2. Establishment of Combustion Kinetics Model

After obtaining the TGA experimental data, the combustion process of oil shale was systematically fitted and analyzed using three kinetic models. The single-step reaction model assumes that the combustion process can be represented by a single activation energy. The distributed activation energy model (DAEM) quantitatively describes complex combustion behavior through a distribution of activation energies. The multi-step reaction model sequentially captures the pyrolysis, organic matter oxidation, and mineral decomposition stages.

Model parameters, including activation energy and frequency factor, were determined using the Ozawa–Flynn–Wall (OFW) method and the Kissinger method. Comparative analysis was conducted to evaluate the fitting accuracy of each model, with particular emphasis on their applicability to different combustion stages.

The Criado method is a method used to analyze non-isothermal reaction kinetics parameters [20]. It normalizes experimental data at different heating rates to the same level of conversion rate, thus drawing the main graph and solving kinetic parameters such as activation energy and frequency factor, described in the following formula:

(1) $$Z\left(\alpha \right)=\left(\frac{d_{\alpha }}{d_{t}}\right)\cdot \frac{T}{\beta }\cdot \exp\left(\frac{E_{\alpha }}{RT}\right)$$

where *α*: degree of conversion, *β*: heating rate, *A*: pre-exponential factor.

The Criado method uses TG and DTG curve data to select the ratio of reaction rate (dα/dt) to reference point (such as α = 0.5) at different conversion rates (α) and combines the integral form G(α) of the theoretical kinetic model function f(α) to plot the relationship curve between [G(α)/G(0.5)] and α, matching it with the standard theoretical curve to determine the optimal reaction mechanism function. Combined with the Arrhenius formula, the apparent activation energy Ea is obtained by linearly fitting ln (dα/dt) to the slope of 1/T, and the pre-exponential factor A is calculated by combining the intercept to obtain the kinetic parameters.

In order to eliminate the interference of dehydration and mineral decomposition stages, we used the Ozawa–Flynn–Wall method and the Kissinger method to calculate the kinetic parameters of this stage based on the main combustion peak (200–600 °C) of the DTG curve.

### 2.3. Data Analysis and Characterization

The key combustion characteristic parameters, including ignition temperature (*T_i_*), peak temperature (*T_p_*), and burnout temperature (*T_f_*), were systematically extracted from thermogravimetric (TG) and derivative thermogravimetric (DTG) curves. Using the TGA experimental data and multi-stage kinetic models, activation energy (Ea) and reaction order (n) were quantitatively calculated through the Kissinger–Arrhenius method and Coats–Redfern analysis. Subsequently, the surface morphology of combustion residues was characterized using scanning electron microscopy (SEM), while the mineralogical composition evolution was determined through an X-ray diffraction (XRD) analysis of residual phases.

### 2.4. Experimental Design Workflow

**Figure 1:**
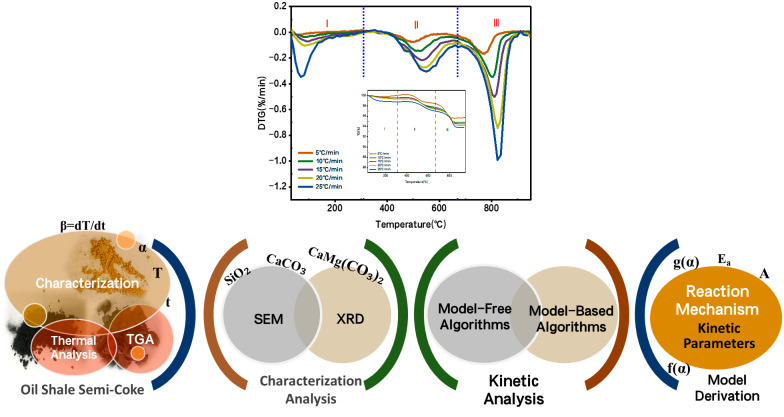
Experimental workflow diagram. Note: I is the dehydration stage; II is the main combustion stage; III is the stage of mineral decomposition.

The experimental workflow is shown in Figure 1.

Firstly, the samples were prepared, and then thermogravimetric analysis (TGA) experiments were carried out. Then, the experimental data were fitted to calculate the relevant kinetic parameters. Finally, the combustion residue was characterized.

## 3. Results and Discussion

### 3.1. Thermogravimetric Analysis (TGA) of Oil Shale

**Figure 2:**
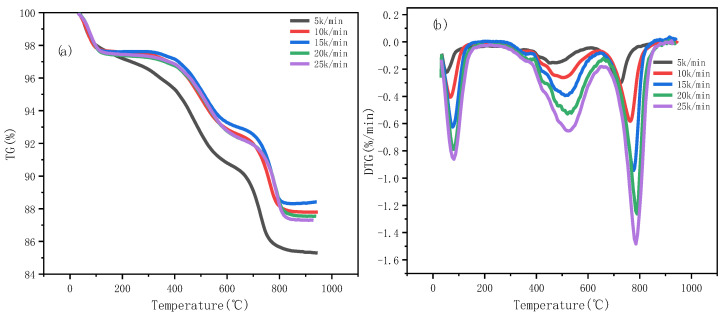
TG–DTG curves of oil shale at different heating rates: (**a**) TG curves, (**b**) DTG curves.

The combustion process of oil shale was systematically studied through thermogravimetric analysis (TGA) experiments, obtaining thermogravimetric (TG) and derivative thermogravimetric (DTG) curves under different heating rates (5 °C/min, 10 °C/min, 15 °C/min, 20 °C/min, and 25 °C/min). The results are shown in Figure 2.

The experiments reveal distinct multi-stage mass loss behaviors, which are closely associated with the decomposition of organic matter and the reactions of mineral components in the oil shale.

#### 3.1.1. Analysis of Thermogravimetric (TG) Curves

The TG curves reveal that the combustion process of oil shale mainly consists of three stages:

(1) Dehydration Stage (50–200 °C): In this stage, the primary mass loss is due to the evaporation of physically adsorbed water and a small amount of chemically bound water. The mass loss ratio is approximately 2–5%. As the heating rate increases, the initial temperature of the dehydration stage slightly rises.
(2) Main Combustion Stage (200–600 °C): This stage accounts for 70–80% of the total mass loss and represents the primary mass loss phase in the oil shale combustion process. The reactions in this stage include the pyrolysis and oxidation of organic matter. The DTG curve shows one or more significant mass loss peaks in this stage, indicating the complex decomposition reactions of organic components. As the heating rate increases, the temperatures corresponding to the mass loss peaks shift toward the higher temperature region (as shown in Table 1), demonstrating that the combustion reaction rate is highly influenced by the heating rate.
(3) Mineral Decomposition Stage (600–900 °C): During this stage, the decomposition of carbonate minerals (e.g., calcite) becomes the dominant reaction, accompanied by the release of CO_2_. Ifticene et al. demonstrated that mineral components (e.g., calcite) in oil shale act as catalysts during combustion, accelerating the decomposition of organic matter and altering the reaction pathway [21]. The mass loss ratio in this stage is approximately 10–15%, and the temperature range and mass loss rate are significantly affected by the mineral composition of the sample.
(4) Residual Stage (above 900 °C): In this stage, the mass change is minimal, primarily due to the presence of stable minerals (e.g., oxides) that do not participate in further reactions.

#### 3.1.2. Analysis of Derivative Thermogravimetric (DTG) Curves

The DTG curves provide characteristic information on the rate of mass loss during combustion: First Peak (Low-Temperature Mass Loss Peak): Corresponding to the dehydration stage, the peak temperature is approximately 50–200 °C. The peak intensity slightly decreases with an increase in the heating rate. Second Peak (High-Temperature Mass Loss Peak): Corresponding to the main combustion stage, the peak temperature range is 200–600 °C, which aligns with the primary oxidation reactions of the organic matter. As the heating rate increases, the peak temperature increases significantly; however, the peak area does not show notable changes. Third Peak (High-Temperature Mineral Decomposition Peak): Corresponding to the mineral decomposition stage, the peak temperature range is 600–900 °C. The position of this peak is closely related to the mineral composition, indicating that mineral decomposition plays an important role in the combustion characteristics of oil shale.

#### 3.1.3. Extraction of Combustion Characteristic Parameters

The key combustion characteristic parameters extracted from the TG and DTG curves are presented in Table 2.

From Table 2, it can be observed that as the heating rate increases, all characteristic temperatures (ignition temperature *T_i_*, peak temperature *T_p_*, and burnout temperature *T_f_*) rise significantly, indicating that the reaction process is strongly influenced by the heating rate. The total mass loss slightly decreases with increasing heating rates, suggesting that higher heating rates may limit the completeness of the reaction. This also reflects the multi-stage nature of oil shale combustion, which includes low-temperature volatile release, organic matter pyrolysis, and mineral decomposition.

### 3.2. Kinetic Analysis

#### 3.2.1. Iso-Conversional Method

The iso-conversional method is a commonly used thermal analysis technique for calculating kinetic parameters, such as activation energy (*E_a_*) and pre-exponential factor (*A*), under fixed conversion rates (*α*) to avoid errors arising from reaction model assumptions. In this study, the Ozawa–Flynn–Wall (OFW) method and the Kissinger method were applied to analyze the combustion kinetics of oil shale.

(1) Theoretical Basis.

In thermogravimetric experiments, the reaction conversion rate *α* is defined as follows:

(2) $$\alpha =\frac{m_{0}-m_{t}}{m_{0}-m_{\infty }}$$

where *m_0_*: initial mass of the sample. *m_t_*: sample mass at a given time. *m_∞_*: final sample mass after the reaction is complete.

The reaction rate equation can be expressed as follows:

(3) $$\frac{d\alpha }{dt}=k(T)f(a)$$

Under non-isothermal conditions, the heating rate is as follows:

(4) $$\beta =\frac{dT}{dt}$$

The reaction rate equation can then be rewritten as follows:

(5) $$\frac{d\alpha }{dT}=\frac{k(T)f(a)}{\beta }$$

By integrating this equation, the relationship between the reaction rate and activation energy is derived as follows:

(6) $$g(\alpha )=\int_{T_{0}}^{T}\frac{A}{\beta }e^{-\frac{Ea}{RT}}dT$$

(2) Ozawa–Flynn–Wall (OFW) Method.

The OFW method does not require assumptions about the reaction mechanism [22]. It calculates the activation energy *E_a_* by plotting ln*β* against 1/*T* for a fixed conversion rate (*α*) using linear regression. The fundamental formula is as follows:

(7) $$\ln\left(\beta \right)=\ln\left[\frac{AE_{a}}{Rg(\alpha )}\right]-5.331-1.052\left(\frac{E_{a}}{RT}\right)$$

In this experiment, the TGA data for different heating rates (5 K/min, 10 K/min, 15 K/min, 20 K/min, and 25 K/min) were used to extract the temperatures corresponding to specific conversion rates (α = 0.1, 0.3, 0.5, 0.7, 0.9). A plot of ln*β* versus 1/*T* was constructed, and activation energy values were calculated for each conversion rate.

The linear relationship between ln*β* and 1/*T* for different conversion rates indicates the strong applicability of the OFW method in this experiment. The results show that the activation energy *E_a_* exhibits a nonlinear trend with respect to the conversion rate, reflecting the complexity of the oil shale combustion process. The detailed results are presented in Table 3.

The nonlinear variation of activation energy indicates that the combustion process of oil shale involves a multi-step reaction mechanism, with different dominant reactions at various stages requiring different energy inputs.

This study conducted a kinetic analysis on the main combustion peak (II *T_p_*), which is consistent with the treatment of the S2 peak in traditional Rock Eval analysis. The average activation energy for this stage was obtained as 143.7 kJ/mol by applying the Ozawa–Flynn–Wall (OFW) method. These results are consistent with the typical activation energy values reported in the literature for organic matter oxidation reactions in oil shale [21,23], thus verifying the effectiveness and specificity of our method.

(3) Kissinger Method.

The Kissinger method is a kinetic calculation approach based on peak temperature (*Tp*) [24], as described by the following formula:

(8) $$\ln\left(\frac{\beta }{{T_{p}}^{2}}\right)=\ln\left(\frac{AR}{E_{a}}\right)-\frac{E_{a}}{R}\times \frac{1}{T_{p}}$$

where *β*: heating rate, *T_p_*: peak temperature.

Using the DTG curves at different heating rates, the corresponding *T_p_* values were extracted for each heating rate. A plot of ln (*β*/*T_p_*^2^) versus 1/T*_p_* was created, and linear regression was performed to calculate the activation energy *E_a_*. From the peak temperature data of the main combustion stage, the activation energy was calculated as *E_a_* = 86.2 kJ/mol, which is close to the result obtained by using the OFW method near *α* = 0.5. The detailed results are presented in Table 4.

The OFW method reveals the trend of activation energy variation with conversion rates during the combustion process of oil shale, highlighting the complexity of the reaction. The Kissinger method provides the average activation energy for the main combustion stage, which validates the results of the OFW method. The combined use of different kinetic calculation methods enhances the reliability of the kinetic parameters and provides an essential basis for modeling the combustion reaction of oil shale. The iso-conversional analysis not only clarifies the energy requirements at different stages of oil shale combustion but also offers theoretical support for optimizing combustion process design.

This study specifically investigated the kinetic characteristics of the primary combustion stage (Peak II *T_p_*) in oil shale, employing an analytical approach analogous to the S2 peak evaluation in conventional Rock–Eval pyrolysis. The Kissinger analysis yielded an average activation energy of 142.94 kJ/mol for this dominant combustion phase, which aligns well with established literature values for organic matter oxidation in oil shale [21,23].

#### 3.2.2. Comparison of Kinetic Calculation Results

**Figure 3:**
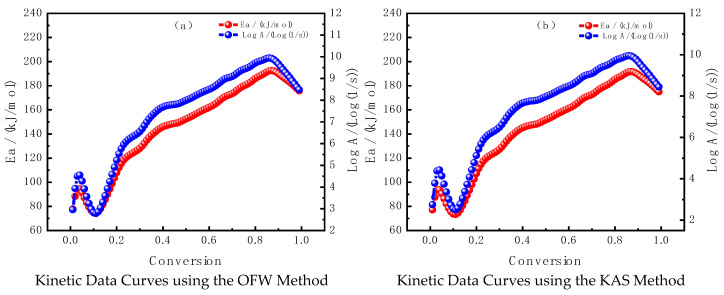
Changes in the apparent activation energy and pre-exponential factor of the main combustion peak during the combustion process of oil shale. Note: The scope of the analysis is limited to the main combustion peak of DTG (200–600 °C), excluding interference from low/high temperature stages.

In order to study the reaction mechanism and energy characteristics during the sample pyrolysis process, and to eliminate the interference of dehydration and mineral decomposition stages, the main combustion peak (200–600 °C) based on differential thermogravimetric (DTG) curves was calculated using the Ozawa–Flynn–Wall (OFW) and Kissinger Akahira Sunose (KAS) methods, and the kinetic parameters of this stage were analyzed. The changes in activation energy and logarithmic frequency factor at different conversion rates were also analyzed. The result is shown in Figure 3.

From the kinetic data curves obtained using the Ozawa–Flynn–Wall (OFW) method in Figure 3a, it is evident that the activation energy (Ea) exhibits significant fluctuations as the conversion rate (α) increases, characterized by multiple peaks and valleys. This suggests that different stages of the oil shale combustion process likely involve distinct reaction mechanisms or physicochemical behaviors, such as volatile release and char combustion. The wide range of fluctuations may reflect atypical reaction behavior at certain stages. The variation of the pre-exponential factor (logA) mirrors that of Ea, indicating a coupled relationship between the two parameters. This coupling aligns with the general correlation between logA and Ea in kinetic models. The observed fluctuations suggest that changes in logA correspond to shifts in the reaction mechanism, further emphasizing the complexity of the combustion process.

In Figure 3b, which presents the kinetic data curves using the Kissinger–Akahira-Sunose (KAS) method, the overall trend of Ea is similar to that observed with the OFW method, also exhibiting fluctuations as the conversion rate increases. However, compared to the OFW method, the amplitude of these fluctuations is smaller, and the curve appears smoother. This suggests that the KAS method may be more stable or less sensitive to external disturbances when processing the data. The variation in the pre-exponential factor (logA) follows a similar trend to Ea, displaying fluctuations with increasing conversion rate, although the amplitude of these fluctuations is less pronounced than that observed with the OFW method. The smoother and more continuous nature of the KAS curves indicates that this method is more suitable for describing overall trends rather than capturing finer details.

Both methods reveal significant fluctuations in Ea and logA as the conversion rate increases, reflecting the complex combustion mechanisms of oil shale. These results suggest that the combustion and pyrolysis processes of oil shale involve a multi-stage reaction mechanism. In the low conversion range (e.g., 0–0.3), the activation energy is relatively low and varies smoothly, corresponding to the release of volatiles, a kinetically simpler phase. In the medium to high conversion range (e.g., 0.4–0.8), Ea fluctuates significantly, accompanied by corresponding changes in logA. This likely reflects the complex nature of char combustion, which may involve multiple sub-stages, such as surface oxidation and gas diffusion. These stages are influenced by various physicochemical factors. The anomalous behavior of activation energy, characterized by significant fluctuations and even negative values at certain stages (e.g., the trough near α = 0.6 in the OFW curve), may be attributed to the interaction of minerals, such as carbonates or metal oxides, within the oil shale. These minerals may catalyze certain reactions or alter the pyrolysis behavior, thereby adding complexity to the process. The OFW method, with its higher fluctuation sensitivity, potentially captures more detailed kinetic behavior, whereas the KAS method produces smoother curves, making it better suited for illustrating overall trends.

**Figure 4:**
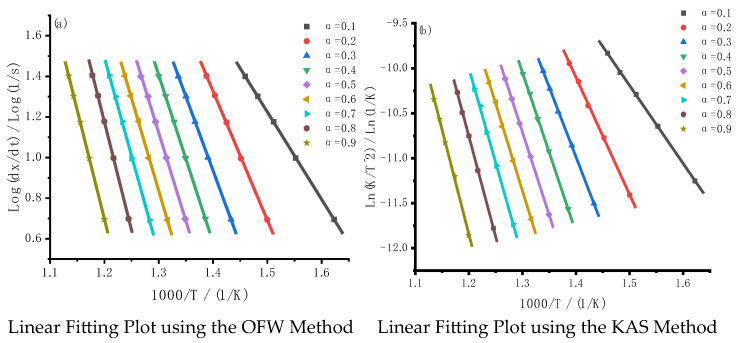
Calculation of oil shale kinetic parameters based on the OFW and KAS methods. Note: The scope of the analysis is limited to the main combustion peak of DTG (200–600 °C), excluding interference from low/high temperature stages.

In experiments with different heating rates, the relationship between Log(dx/dt) and 1000/T at the same conversion rate is shown in Figure 4. This figure highlights the linearity of the relationship for the iso-conversional methods and serves as a key foundation for extracting reliable kinetic parameters.

The relationship between Log(dx/dt) and 1000/T at various conversion rates for the oil shale sample at different heating rates is shown in Figure 4. Each iso-conversion curve corresponds to the kinetic behavior at different conversion rates (α = 0.1 to α = 0.9), representing distinct stages in the combustion and pyrolysis processes. As the conversion rate increases, the slope of the curves changes, highlighting significant variations in kinetic behavior at different stages of the reaction.

The slope of these curves is directly related to the activation energy (Ea). As the conversion rate increases, the slope of the curve increases, indicating that the activation energy rises as the reaction progresses. This suggests that, in the low conversion rate stage (α = 0.1 to 0.3), the activation energy is low, and the combustion process is likely dominated by the release of easily decomposable volatiles. The reactions are relatively simple, requiring less energy. Medium Conversion Rate Stage (α = 0.4 to 0.6): As the conversion rate increases, the reaction becomes more complex, transitioning to char decomposition and combustion. The activation energy rises significantly as the reactions involve more intricate processes. High Conversion Rate Stage (α = 0.7 to 0.9): At this stage, char combustion is nearly complete, and residual minerals may participate in reactions or undergo surface diffusion and gas transfer processes. These contribute to the higher activation energy observed at this stage.

Oil shale contains various organic and inorganic components with different decomposition temperatures and reaction complexities, which lead to significant changes in activation energy across different stages. Additionally, gas–solid coupled reactions and mineral catalytic effects during combustion further contribute to the variation in activation energy across these stages.

The increase in activation energy with conversion rate indicates that the combustion and pyrolysis processes of oil shale are not governed by a single mechanism but are instead composed of multiple stages, each with its own kinetic characteristics. These stages involve (1) the rapid release and combustion of volatiles (low activation energy), (2) the combustion of solid char (high activation energy), and (3) the thermal decomposition or catalytic effects of minerals (stage-specific activation energy changes). The iso-conversion curves and fluctuations in activation energy, as depicted in the figure, highlight the complexity and multi-stage nature of the oil shale combustion and pyrolysis processes.

This complexity arises from the multi-component nature of the material and the involvement of multiple mechanisms in the combustion process. The trend of increasing activation energy with conversion rate reinforces the idea that the combustion and pyrolysis of oil shale occur through complex, multi-stage reactions with distinct kinetic behaviors at each stage. The low conversion rate stage is dominated by the release and combustion of volatiles, while the high conversion rate stage primarily involves char decomposition and combustion, alongside mineral participation.

The kinetic data calculated based on the OFW and KAS model-free methods are shown in Table 5.

Table 5 presents the kinetic parameters (activation energy Ea and frequency factor logA) for oil shale combustion at different conversion rates (α) calculated using the OFW (Ozawa–Flynn–Wall) method and KAS (Kissinger) method.

The activation energy (Ea) obtained using the OFW method exhibits a fluctuating trend with increasing conversion rates: At α = 0.1, Ea is 74.7 kJ/mol, which is relatively low, indicating that the initial stage (volatile release stage) involves relatively easy reactions. As the conversion rate increases, the activation energy rises steadily and reaches a maximum of Ea = 145.9 kJ/mol at α = 0.4, indicating that this stage is dominated by the combustion of solid char, which requires higher activation energy. Subsequently, the activation energy decreases slightly but remains at a high level (Ea ≈ 152.4–162.0 kJ/mol), likely associated with mineral interactions or residual reactions. The activation energy (Ea) calculated using the KAS method shows a similar trend to the OFW method, with comparable values but slightly smaller, especially at low conversion rates. At α = 0.6, the maximum activation energy of Ea = 162.0 kJ/mol is consistent between the two methods, demonstrating good compatibility between the results of the OFW and KAS methods.

The frequency factor (A) is relatively low in the low conversion rate stage (α = 0.1 to 0.3), reflecting a low reaction rate, likely due to the release of volatiles. As the conversion rate increases, A also increases, peaking around α = 0.5–0.6 (OFW method: 1.00 × 10^10^ s^−1^, KAS method: 3.16 × 10^10^ s^−1^). This indicates that molecular collision frequency significantly increases during the char combustion stage. In the high conversion rate stage (α = 0.7 to 0.9), logA decreases again, suggesting that the reaction stabilizes or enters the residual combustion stage. Both methods show consistent trends in the variation of activation energy (Ea) and frequency factor (logA) with conversion rate, with both increasing and then decreasing as the conversion rate rises. This reflects the multi-stage nature of the combustion process. At a critical stage (α = 0.6), the results from the two methods are highly consistent (Ea = 162.0 kJ/mol). At low conversion rates (α = 0.1), the activation energy calculated using the OFW method (74.7 kJ/mol) is slightly higher than that of the KAS method (73.7 kJ/mol). The frequency factor (A) calculated using the OFW method is slightly higher than that of the KAS method, suggesting that the OFW method may be more sensitive to molecular collision frequency at higher temperature ranges. Low conversion rate stage (α = 0.1–0.3): The activation energy is low, and the frequency factor is small, reflecting the relatively simple reactions during the volatile release stage, where the reaction rate is low. Medium conversion rate stage (α = 0.4–0.6): The activation energy rises significantly, and the frequency factor reaches a peak, reflecting the intense and complex reactions during the char combustion stage, which requires higher energy input. Regarding the high conversion rate stage (α = 0.7–0.9), the activation energy slightly decreases but remains at a high level, while the frequency factor decreases. This stage is likely related to mineral participation or the combustion of residues.

The fluctuations in activation energy with conversion rate indicate the significant multi-stage characteristics of the oil shale combustion process, primarily including volatile release, char combustion, and residual reactions. The results of the OFW and KAS methods show good consistency and effectively describe the kinetic behavior of the oil shale combustion process. Combining the results of both methods provides a more comprehensive understanding of the reaction mechanisms and kinetic characteristics at each stage of oil shale combustion, offering valuable data support for optimizing the combustion process.

#### 3.2.3. Traditional Mechanism Model Solutions

**Figure 5:**
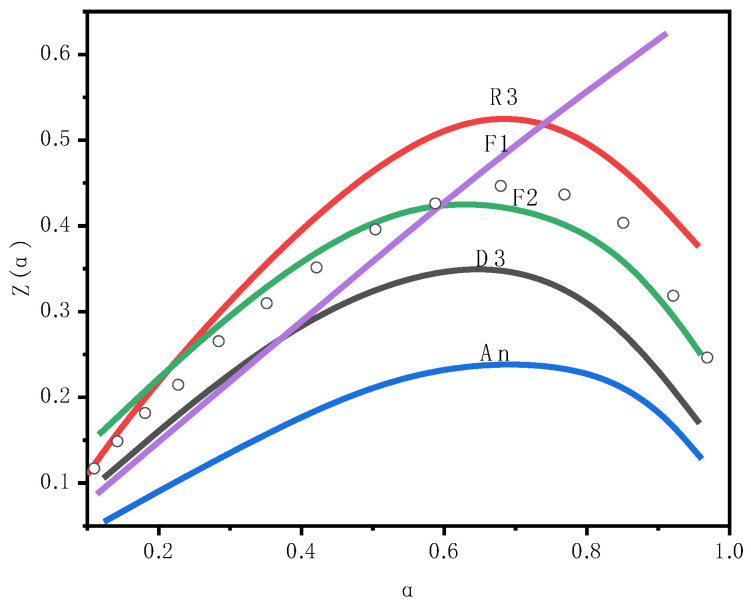
The master curve Z(α). The experimental data converted from Equation (1).

The value of the Z(α) function calculated using Equation (1) is shown as the hollow circle in Figure 5. Obviously, the experimental data are in good agreement with the main curve of the F2 mechanism. By comparing the Z(α) curve obtained from the experiment with theoretical curves of different reaction mechanisms, the most suitable reaction mechanism for describing the combustion process of oil shale can be determined. In this study, the experimental data matched well with the main curve of the F2 mechanism (second-order reaction), indicating that the dynamic behavior of the main combustion stage of oil shale conforms to the second-order reaction mechanism. The F2 model (second-order reaction model) assumes that the reaction rate is proportional to the square of the reactant concentration (i.e., reaction order n = 2), and this mechanism is typically applicable to reactions involving bimolecular collisions or surface adsorption control.

To identify the mechanism model that best fits the experimental data, traditional pyrolysis kinetic theory was applied. It was assumed that the pyrolysis process could be divided into multiple reaction stages (e.g., Fn, D3, An, etc.), and the kinetic mechanism function G(α) was used, along with reaction kinetic parameters (e.g., activation energy Ea, reaction order n, frequency factor logA) for fitting. Table 6 presents the kinetic data results for the multi-step model.

From Table 6, the models listed can be categorized into three main types: diffusion-controlled models, nucleation and growth-controlled models, and reaction-controlled models. By combining the kinetic parameters of each stage in Table 5, the mechanisms can be analyzed as follows:

(1) Multi-Step Reaction Models.

The table lists various reaction models and their corresponding step combinations, including the Fn-Fn-Fn model, An-D3-R3 model, D3-R3-An model, and R3-An-D3 model. Each model is divided into multiple steps (e.g., “Step 1, Step 2, Step 3”), reflecting the complexity of the combustion process, which requires multi-step reaction models for accurate descriptions.

(2) Characteristics of Activation Energy (Ea) and Frequency Factor (A).

Fn-Fn-Fn Model: Activation energy increases from 45.5 kJ/mol in Step 1 to 114.2 kJ/mol in Step 3, reflecting the increasing complexity of the combustion process. The low activation energy steps likely correspond to volatile release, while the high activation energy steps correspond to char combustion. An-D3-R3 Model: Activation energy is very high in Step 1 (155.7 kJ/mol) and Step 2 (352.4 kJ/mol), indicating that this model may describe more complex reactions. In Step 3, the activation energy decreases to 109.1 kJ/mol, possibly corresponding to the late-stage reactions of residual materials. D3-R3-An and R3-An-D3 Models: These models exhibit lower overall activation energies (e.g., the maximum activation energy in the R3-An-D3 model is 72.9 kJ/mol), making them more suitable for describing volatile release or simpler combustion processes.

The frequency factor (A) is correlated with activation energy, with steps with higher activation energy typically corresponding to larger A values. For example, in Step 2 of the An-D3-R3 model, the activation energy is as high as 352.4 kJ/mol, and the frequency factor reaches 2.02 × 10^12^ s^−1^. In contrast, the frequency factor in the R3-An-D3 model is generally lower, reflecting simpler reaction pathways.

(3) Variation in Reaction Order (n).

The reaction order in different steps indicates the complexity of the reaction and the influence of reactant concentrations on the reaction rate. Fn-Fn-Fn Model: The reaction order in Step 1 is relatively high (n = 1.983), although it decreases in Step 2 and Step 3 (n = 0.797), indicating a reduction in reaction complexity.

(4) Step Contribution.

Fn-Fn-Fn Model: Step 3 has the highest contribution (0.495), indicating that this stage dominates the overall reaction process. An-D3-R3 Model: Step 3 also has the highest contribution (0.455), suggesting that the late-stage reactions play a significant role in the overall process. R3-An-D3 Model: Step 3 has an even higher contribution (0.632), highlighting the importance of residual material combustion in this model.

Different models are suited to describe different combustion stages. For example, the Fn-Fn-Fn model is suitable for describing simple volatile release and char combustion processes. The An-D3-R3 model is better suited for more complex reaction pathways. The variations in activation energy reflect the multi-stage nature of the combustion process. The activation energy is lower during the volatile release stage, while it is higher during char combustion and reactions involving mineral components. Based on R^2^ values and step contributions, the Fn-Fn-Fn model and An-D3-R3 model exhibit higher applicability and can be used to explain the key kinetic characteristics of the oil shale combustion process. The data in this table provide important theoretical support for the further optimization of oil shale combustion efficiency and for understanding its reaction mechanisms.

(5) Reaction Characteristics and Control Mechanisms at Each Stage.

By combining commonly used models and kinetic parameters from Table 6, the control mechanisms and mechanistic characteristics of different stages are summarized in Table 7.

From Table 7, the control models and reaction mechanisms of combustion reactions at different stages can be summarized as follows:

(1) Volatile Release Stage.

This is the initial stage, primarily involving the rapid release of volatiles (light hydrocarbons and small-molecule gases). The pyrolysis rate dominates, with relatively simple reactions that are primarily physical and chemical decomposition processes. The chemical reaction rate is controlled, while mass transfer effects are weak. Kinetic Parameters: Activation energy is low, e.g., in the Fn-Fn-Fn model, Ea = 45.5 kJ/mol. The frequency factor is small, e.g., logA = 1.138 (Fn-Fn-Fn model) or negative (R3-An-D3 model).

The reaction order is relatively high, e.g., n = 1.983 in the Fn-Fn-Fn model, indicating that the reaction rate is concentration-dependent. This stage involves the combined physical and chemical processes of devolatilization.

(2) Char Combustion Stage.

This is the intermediate stage, where the dominant reactions involve the oxidation and decomposition of solid char, releasing significant amounts of energy. The reaction complexity increases due to the coupling of chemical reaction rates and mass transfer effects. This stage may also be influenced by mineral catalytic or inhibitory effects.

Kinetic Parameters: Activation energy increases significantly, e.g., in Step 2 of the An-D3-R3 model, Ea = 352.4 kJ/mol. The frequency factor is large, e.g., logA = 20.103 (An-D3-R3 model), indicating high molecular collision frequency. The reaction order is medium to high, e.g., n = 9.000 in the An-D3-R3 model, reflecting the complexity of the combustion mechanism. Char combustion involves gas-solid reactions, which may include surface oxidation, gas diffusion, and the formation of oxides.

(3) Residue Reaction Stage.

This is the final stage, mainly involving the slow reactions of mineral components and refractory organic materials. Chemical reactions gradually weaken, while diffusion and mass transfer effects become dominant. Residue combustion may be governed by diffusion limitations. Kinetic Parameters: Activation energy is moderate, e.g., in Step 3 of the R3-An-D3 model, Ea = 72.9 kJ/mol. The frequency factor is relatively low, e.g., logA = −0.032 (D3-R3-An model). The reaction order is low, e.g., n = 0.348 in the D3-R3-An model, indicating weak dependency on chemical reactions.

This stage may involve mineral catalytic decomposition, slow oxidation of residual carbon, and the thermal decomposition of carbonates and other inorganic materials.

Fn-Fn-Fn Model: Suitable for describing volatile release and the initial combustion stage. An-D3-R3 Model: Suitable for complex reaction stages, especially during char combustion. D3-R3-An Model: More suitable for simple combustion processes, such as the volatile release stage. R3-An-D3 Model: Applicable to the residue combustion stage, describing the oxidation of refractory components.

**Figure 6:**
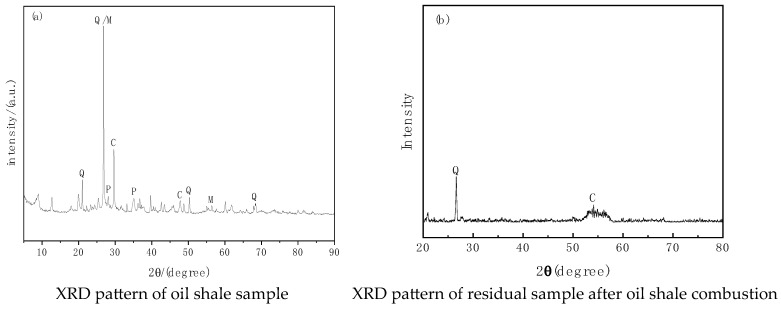
XRD patterns of oil shale and residual samples after combustion. Note: C—calcite, M—dolomite, P—pyrite, Q—quartz.

Through multi-stage model fitting (Fn-Fn-Fn), it was found that the activation energy Ea of each stage is consistent with the trend of acid treated kerogen in the literature [15], especially the Ea value of the main combustion stage (α = 0.3–0.7) is 206–223 kJ/mol, which is consistent with the oxidation activation energy of acid treated kerogen (200–220 kJ/mol). Although the cheese roots were not separated, combined with multi-stage models and mineral XRD/SEM analysis (Figures 6 and 9), we confirmed the interaction between organic matter and minerals and pointed out that this interaction is consistent with the kinetic trend of acid-treated samples in reference [3].

#### 3.2.4. Comparison of Model-Free and Model-Based Methods

In the analysis of oil shale combustion kinetics, traditional reaction models and model-free methods can complement each other. Model-free methods are more suitable for rapid estimation of activation energy and overall trend analysis, while traditional models can provide detailed reaction order and rate information within specific intervals. By combining the two approaches, a more comprehensive understanding of the complex pyrolysis kinetics of oil shale can be achieved, providing theoretical support for the practical application of thermal recovery technologies. Table 8 presents a comparative analysis of model-free and model-based methods.

From Table 8, the comparison between model-free and model-based methods reveals the following insights: The activation energy calculated using model-free methods fluctuates with the conversion rate (e.g., as shown in Table 6, the **Ea** values from the OFW and KAS methods range between 27.8 kJ/mol and 245.0 kJ/mol), making them suitable for analyzing overall combustion trends. Model-based methods calculate activation energy for each stage; for example, Step 2 of the An-D3-R3 model has Ea = 352.4 kJ/mol, which is significantly higher and reflects the precise characteristics of complex reactions.

The frequency factor calculated using model-free methods is strongly correlated with activation energy but lacks stage-specific differentiation (e.g., as shown in Table 6, the variation in logA from the OFW and KAS methods is relatively small). In contrast, the frequency factor distribution in model-based methods shows significant stage-specific differences (e.g., in Step 2 of the An-D3-R3 model, logA = 20.103, which is much higher than in other steps, reflecting the collision frequency requirements of high-temperature complex reactions).

Model-free methods describe the combustion process solely through the conversion rate, making it difficult to clearly distinguish specific stages (e.g., the boundary between volatile release and char combustion is unclear). In contrast, model-based methods clearly define stages (e.g., the three-step model in Table 4: volatile release, char combustion, and residue reactions) and can quantify the contribution of each stage (e.g., Step 3 in the R3-An-D3 model contributes 0.632). According to the Criado method, the main combustion stage follows a second-order reaction (F2) mechanism. The revised pre-exponential factor has decreased by approximately 0.5 log units, indicating that the original model may have overestimated the frequency of molecular collisions. This discovery is consistent with the mineral catalytic effect, which means that the actual energy barrier has been reduced [20].

Model-free methods are relatively simple to calculate and are suitable for quickly evaluating combustion kinetic parameters. Model-based methods, on the other hand, are more complex and require experimental fitting combined with mechanistic assumptions, making them suitable for detailed mechanistic studies and combustion optimization. Model-free methods are ideal for quickly obtaining global kinetic parameters and for preliminary evaluations of combustion characteristics. Model-based methods are more precise and can provide detailed insights into the characteristics and mechanisms of specific combustion stages, making them an ideal tool for in-depth studies of combustion mechanisms and optimization of combustion processes. The two methods can be used in combination: model-free methods can be used to quickly evaluate kinetic parameters, followed by model-based methods for in-depth analysis of the control mechanisms at different stages, enabling a comprehensive understanding of the oil shale combustion process.

### 3.3. Surface Morphology and Mineral Composition of Oil Shale Before and After Combustion

To reveal the changes in mineral composition and crystalline structure of residual materials after combustion, X-ray diffraction (XRD) analysis was performed on oil shale and combustion residue samples. The results are shown in Figure 6.

From Figure 6, it can be observed that the main mineral phases in the oil shale sample (Figure 6a) include a significant amount of minerals such as silicates (quartz), carbonates (calcite and dolomite), and sulfides (pyrite). These minerals provide the oil shale with a mineral framework and serve as a basis for chemical reactions. The presence of pyrite may result in the production of sulfur dioxide (SO_2_) during combustion, which poses challenges for the treatment of combustion flue gases.

In the combustion residue (Figure 6b), the diffraction peaks of quartz remain evident, indicating that quartz has strong thermal stability and does not decompose at high temperatures. The calcite peaks are significantly weakened, suggesting that part of the calcite decomposed during combustion, possibly forming calcium oxide (CaO). During combustion, some pyrite and carbonate minerals undergo high-temperature decomposition:

1. Pyrite decomposition:

FeS_2_ + O_2_ → Fe_2_O_3_ + SO_2_↑

This reaction produces iron oxide and sulfur dioxide, which influence the chemical properties of the residue.

2. Calcite decomposition:

CaCO_3_ → CaO + CO_2_↑

This reaction releases carbon dioxide and forms calcium oxide, which may further react with other substances to form new mineral phases. The diffraction peaks of quartz (Q) remain almost unchanged, indicating its chemical stability and resistance to decomposition during high-temperature combustion. Pyrite undergoes partial decomposition during combustion, producing iron oxides and sulfur dioxide. Carbonate minerals such as calcite and dolomite decompose, releasing CO_2_ and potentially forming calcium oxide. Quartz demonstrates high thermal stability, does not participate in the reactions, and remains as a residual mineral.

**Figure 7:**
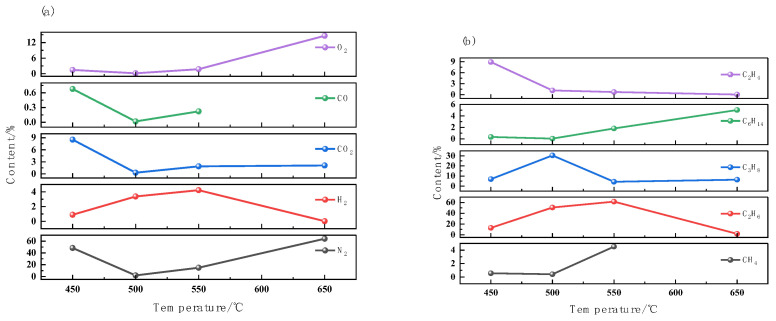
Changes of gas composition after oil shale combustion at different temperatures: (**a**) non-hydrocarbon gas; (**b**) hydrocarbon gas.

To study the generation and variation laws of non-hydrocarbon gases and hydrocarbon gases released during the combustion process of oil shale, an air injection combustion experiment of oil shale was conducted using a high-temperature and high-pressure aluminum reactor, and the evolution of the main non-hydrocarbon gases and hydrocarbon gases during the combustion process was determined. The experimental results are shown in Figure 7.

The experimental data show that the characteristics of gas components changing with temperature can be summarized into the following three dominant reaction mechanisms:

(1) Thermal Dehydrogenation.

Within the temperature range of 500–550 °C, the concentration of H_2_ peaks, indicative of the thermal decomposition and dehydrogenation of organic matter, while the increase in C_2_H_6_ and C_3_H_6_ suggests the scission of carbon-hydrogen bonds.

(2) Hydrocarbon Combustion.

The concentration of O_2_ continues to decrease during the heating process, and the CO/CO_2_ ratio first increases and then decreases, with a peak at 580 °C. This change is consistent with the two-stage oxidation combustion model experienced by hydrocarbons: the initial stage (T < 500 °C) generates CO, and the later stage (T > 600 °C) fully burns to generate CO_2_. These stages are consistent with optimized combustion pathways identified by Zanoni et al. [23], which describe the stepwise transformation of organic matter and minerals during combustion.

(3) Mineral Calcination.

The release of CO_2_ can be attributed to the thermal decomposition of carbonates (such as calcite CaCO_3_). This is also consistent with the XRD analysis of combustion residues, indicating that carbonates and other mineral components undergo chemical changes at high temperatures.

**Figure 8:**
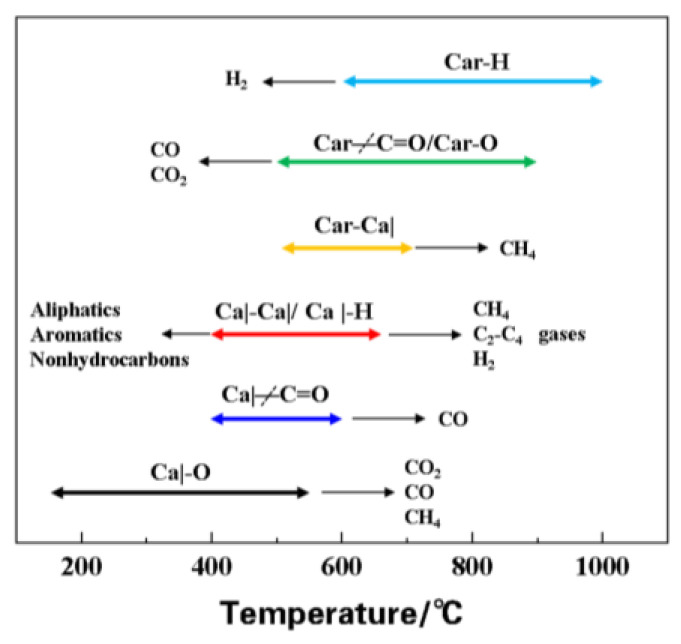
Schematic diagram of the main reactions that occur in oil shale at different temperature ranges [25].

Figure 8 shows the complex chemical changes that occur in oil shale during a temperature rise. The low-to-medium temperature stage (200–600 °C) mainly involves dehydration and thermal dissociation of organic matter, releasing gases such as H_2_, CO, and CO_2_. During the high-temperature stage (>600 °C), carbonate minerals mainly decompose, producing gases such as CH_4_, C_2_H_4_, and C_4_H_4_. The breakage of C-H and C-C bonds leads to the generation of methane and olefin gases, respectively. These processes reflect the multi-stage characteristics and complex reaction mechanisms of oil shale pyrolysis and combustion, providing theoretical support for optimizing the combustion process.

**Figure 9:**
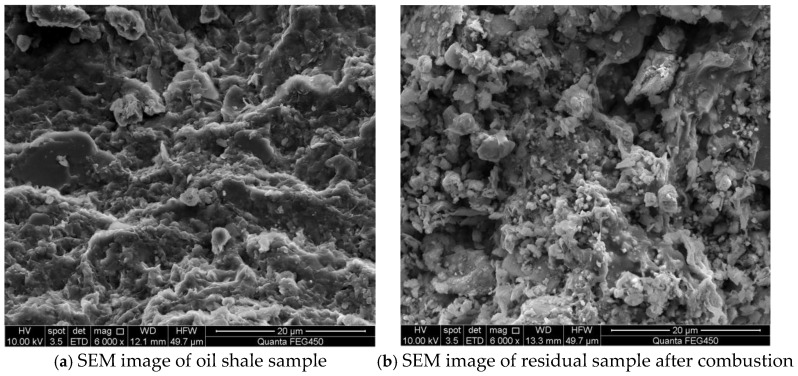
SEM images of oil shale and residual sample after combustion.

From Figure 9, the comparative SEM analysis of oil shale and combustion residue samples reveals the following:

Figure 9a shows a relatively dense and layered structure, indicating that oil shale contains a combination of organic matter and minerals. The surface is smooth but contains some small pores or cracks, which may have been formed due to the presence of organic matter and the influence of external pressure or temperature during its formation. The layered or granular features suggest that oil shale is composed of a mixture of minerals (e.g., quartz, calcite) and organic components.

Figure 9b shows the microstructure of the combustion residue sample, which displays a loose and porous structure, significantly different from the dense structure observed before combustion. The surface of the residue contains many particles and fragments, indicating that minerals decomposed and reorganized during the combustion process. The number of pores increased significantly, and the connectivity between particles weakened, exhibiting the typical characteristics of high-temperature reaction products.

The combustion process led to the complete decomposition of organic matter, along with the high-temperature decomposition of minerals (e.g., calcite decomposing into CaO), making the structure looser and more porous. The porous structure may have been caused by the release of gases (e.g., CO_2_, SO_2_), which is consistent with the mineral decomposition reactions during combustion.

The post-combustion residue is primarily composed of thermally stable minerals (e.g., quartz) and newly formed oxides (e.g., CaO). The weak bonding between these minerals results in a granular appearance of the residue.

Before combustion, the oil shale exhibits a relatively dense layered structure; meanwhile, after combustion, it becomes loose and porous. The high-temperature effects during combustion decompose certain mineral phases (e.g., calcite, pyrite), releasing gases and forming new oxides and particles. The significant increase in the porosity of the combustion residue may affect its further reaction characteristics and utilization value.

## 4. Conclusions

(1) The combustion process can be divided into three main stages: the dehydration stage, the main combustion stage, and the mineral decomposition stage. Among these, the main combustion stage contributes the most (70–80% of mass loss) and is the key phase of the combustion process. In the combustion residue, quartz exhibits strong high-temperature stability, while carbonate minerals (e.g., calcite) and sulfides (e.g., pyrite) decompose, forming oxides and releasing gases.

(2) Iso-conversional methods (e.g., OFW and KAS methods) reveal the fluctuation characteristics of activation energy with conversion rate during combustion, indicating a multi-step reaction mechanism. In model-based methods, the second-order reaction model (Fn, n = 1.98) and diffusion model (D3) are suitable for different reaction stages and can accurately describe the kinetic characteristics of the combustion process. The nonlinear variation of activation energy and frequency factor demonstrates the high complexity of the combustion process, involving multi-stage behaviors such as volatile release, char combustion, and mineral reactions.

(3) XRD and SEM analyses of the combustion residue show that combustion significantly changes the mineral composition and microstructure. The combustion residue exhibits a loose and porous structure. By combining the advantages of model-free and model-based methods, the combustion kinetics of oil shale can be comprehensively characterized, providing scientific guidance for combustion process optimization and equipment design.

(4) This study provides important theoretical support for the efficient utilization of oil shale as an unconventional energy resource and lays the foundation for further development of combustion kinetic models and their industrial applications.

In conclusion, the multi-stage reaction characteristics and kinetic behaviors observed during oil shale combustion provide valuable scientific evidence and practical references for the clean and efficient utilization of unconventional energy resources.

## Figures and Tables

**Table 1:** Elemental composition and mineral content of oil shale samples.

Elemental Analysis (wt%)		Proximate Analysis (wt%)		Mineral Composition (wt%)			
C	13.44	Moisture	3.8	Quartz	28	Dolomite	7.6
H	0.46	Volatile matter	28.4	Feldspar	5.2	Siderite	0.5
N	0.38	Ash	64.2	Clay minerals	23.2	Pyrite	1.7
S	0.58	Fixed carbon	3.6	Calcite	33.8		

**Table 2:** Combustion characteristic parameters at different heating rates.

Heating Rate (°C/min)	*T_i_* (°C)	I *T_p_* (°C)	II *T_p_* (°C)	III *T_p_* (°C)	*T_f_* (°C)	Total Mass Loss (%)
5	343	52	486	708	783	12.32
10	362	79	507	741	801	12.93
15	367	83	508	755	822	11.43
20	388	91	511	762	845	11.21
25	403	95	525	773	867	11.18

**Table 3:** The relationship between conversion rate and activation energy, calculated using the OFW method.

Conversion Rate (*α*)	0.1	0.3	0.5	0.7	0.9
Activation energy (*E_a_*, kJ/mol)	74.7	128.1	152.4	173.4	190.1

**Table 4:** The relationship between the conversion rate and activation energy was calculated by using the Kissinger method.

Conversion Rate (*α*)	0.1	0.3	0.5	0.7	0.9
Activation energy (*E_a_*, kJ/mol)	73.7	127.3	151.7	172.7	189.3

**Table 5:** Kinetic data calculated using the OFW method and KAS model-free method.

Conversion	Ozawa–Flynn–Wall Method		Kissinger Method	
α	Ea/(kJ/mol)	A/(1/s)	Ea/(kJ/mol)	A/(1/s)
0.1	74.7	6.31 × 10^2^	73.7	3.98 × 10^2^
0.2	108.0	1.58 × 10^5^	107.2	1.26 × 10^5^
0.3	128.1	3.98 × 10^6^	127.3	3.16 × 10^6^
0.4	145.9	5.01 × 10^7^	145.1	5.01 × 10^7^
0.5	152.4	1.00 × 10^8^	151.7	1.00 × 10^8^
0.6	162.0	3.16 × 10^8^	161.3	3.16 × 10^8^
0.7	173.4	1.26 × 10^9^	172.7	1.26 × 10^9^
0.8	186.0	5.14 × 10^9^	185.2	5.01 × 10^9^
0.9	190.1	4.69 × 10^9^	189.3	4.69 × 10^9^

**Table 6:** Kinetic data for multi-step models.

	Contribution	React Order n	Ea/(kJ/mol)	A/(1/s)	R^2^
Fn-Fn-Fn					0.9933
Stage 1	0.277	1.983	45.5	1.37 × 10^1^	
Stage 2	0.229	0.797	109.7	1.01 × 10^5^	
Stage 3	0.495	0.865	114.2	1.76 × 10^3^	
An-D3-R3					0.9915
An	0.311	0.302	155.7	4.07 × 10^9^	
D3	0.234	\	352.4	1.27 × 10^20^	
R3	0.455	\	219.9	2.02 × 10^8^	
D3-R3-An					0.9898
D3	0.277	\	39.1	1.62 × 10^−2^	
R3	0.211	\	36.1	6.62 × 10^−1^	
An	0.513	0.348	−1.4	1.26 × 10^−2^	
R3-An-D3					0.9901
R3	0.178	\	22.4	2.25 × 10^−2^	
An	0.193	0.110	101.0	1.22 × 10^5^	
D3	0.632	\	72.9	9.29 × 10^−1^	

**Table 7:** Analysis of control models and reaction mechanisms for combustion reactions at different stages.

Stage	Control Mechanism	Activation Energy (Ea)	Frequency Factor (logA)	Reaction Order (n)	Mechanism
Volatile release stage	Dominated by pyrolysis rate	Low (e.g., Ea = 45.5)	Small (e.g., logA = 1.138)	Relatively high (e.g., n = 1.983)	Physicochemical decomposition, releasing small molecule gases
Coke combustion stage	Coupling of chemical reaction rate and mass transfer	High (e.g., Ea = 352.4)	Large (e.g., logA = 20.103)	Moderate or high (e.g., n = 9.000)	Surface oxidation, gas-phase mass transfer, and gas–solid combustion reaction of coke
Residual reaction stage	Gradual dominance of diffusion and mass transfer	Moderate (e.g., Ea = 72.9)	Small (e.g., logA = −0.032)	Low (e.g., n = 0.3488)	Mineral catalysis, slow oxidation of refractory residual carbon, and decomposition of carbonates

**Table 8:** Comparative analysis of model-free and model-based methods.

Comparison Dimension	Model-Free Methods (OFW, KAS)	Model-Based Methods (Multi-Step Models: Fn-Fn-Fn, An-D3-R3, etc.)
Characteristics of Analytical Methods	Based on integral or differential equations, calculates activation energy (Ea) and frequency factor (logA) without assuming a reaction model.	Assumes specific reaction models (e.g., Fn, D3, etc.), describes each reaction stage step-by-step, and calculates more detailed kinetic parameters (Ea, logA, n).
Stability of Calculation Results	Results are sensitive to fluctuations in reaction stages at different conversion rates, with activation energy (Ea), using the OFW method, showing significant fluctuations with conversion rates (e.g., Table 3 shows noticeable variations).	Results are more stable, with high goodness of fit (e.g., R^2^ ≈ 0.99), and can more accurately reflect the kinetic characteristics of each reaction stage.
Applicability	Suitable for quickly estimating overall kinetic parameters, applicable for preliminary studies of combustion or pyrolysis, especially when materials are complex and reaction pathways are unknown.	Suitable for detailed analysis, particularly for describing multi-stage reaction mechanisms in combustion processes (e.g., volatile release, char combustion, residual combustion).
Activation Energy (Ea)	Activation energy shows significant fluctuations and varies with conversion rates (e.g., Table 3, OFW method: Ea = 27.8–238.6 kJ/mol).	Activation energy is more accurately fitted to specific models (e.g., Fn-Fn-Fn model: Ea = 45.5–114.2 kJ/mol).
Frequency Factor (logA)	Highly correlated with activation energy, showing similar trends and certain fluctuations with increasing conversion rates (e.g., Table 3, OFW method: logA = 10.4–13.3).	Frequency factors have greater physical significance within each model stage (e.g., An-D3-R3 model: logA = 5.005–20.103), aligning more closely with actual reactions.
Reaction Order (n)	Reaction order is not directly calculated, focusing only on kinetic parameters.	Provides reaction orders (e.g., An-D3-R3 model: n = 0.302–9.000), enabling more accurate descriptions of the influence of different reaction stages on reaction rates.
Mechanism-Revealing Capability	Only provides overall trends in kinetic parameters, with limited capability to reveal multi-stage reaction mechanisms.	Clearly describes control mechanisms at different stages (e.g., volatile release, char combustion, residual reaction), offering better explanations for complex reaction pathways.
Goodness of Fit (R^2^)	Does not directly provide goodness of fit, mainly relies on linear relationships to indirectly measure the reliability of results (e.g., trends in Table 3 data).	High goodness of fit (e.g., Table 4, R^2^ ≈ 0.99), effectively evaluating the applicability of reaction models and the reliability of results.
Calculation Complexity	Relatively simple calculations and kinetic parameters can be quickly derived from experimental data, suitable for preliminary research.	Requires assumptions about specific reaction models, involves complex calculation steps and multiple parameters, suitable for in-depth studies of multi-stage complex combustion processes.

## Data Availability

The original contributions presented in this study are included in the article. Further inquiries can be directed to the corresponding author.
